# Comparison of robotic vs laparoscopic left-sided colorectal cancer resections

**DOI:** 10.1007/s11701-022-01414-9

**Published:** 2022-05-24

**Authors:** T. S. Hettiarachchi, A. Askari, E. Rudge, L. T. Hao, S. Sarwar, D. Dowsett, A. El Hadi, Irshad Shaikh

**Affiliations:** 1 Norfolk and Norwich University NHS Foundation Trust, Norwich, UK; 2 Cambridge University Hospitals NHS Foundation Trust, Cambridge, UK

**Keywords:** Robotic surgery, Colon cancer, Laparoscopic surgery, Bowel resection, Cancer, Colorectal surgery

## Abstract

Robotic assisted surgery (RAS) has become increasingly adopted in colorectal cancer surgery. This study aims to compare robotic and laparoscopic approaches to left sided colorectal resections in terms of surgical outcomeswith no formal enhanced recovery programme. All patients undergoing robotic or laparoscopic left sided or rectal (high and low anterior resection) cancer surgery at a single tertiary referral centre over 3 years were included.A total of 184 consecutive patients from July 2017 to December 2020 were included in this study, with 40.2% (n=74/184) undergoing RAS. The median age at time of surgery was 68 years (IQR 60-73 years). RAS had a significantly shorter length of median stay of 3 days, compared to 5 days in the conventional laparoscopic surgery (CLS) group (p<0.001). RAS had a significantly lower rate of conversion to open surgery (0% vs 16.4%, p<0.001). The median operative time was also shorter in RAS (308 minutes), compared to CLS (326 minutes, p=0.019). The overall rate of any complication was 16.8%, with the RAS experiencing a lower complication rate (12.2% vs 20.0%, p=0.041). There was no significant difference in anastomotic leak rates between the two groups (4.0% vs 5.5%, p=0.673), or in terms of complete resection (R0) (robotic 98.6%, laparoscopic 100%, p=0.095). Robotic left sided colorectal surgery delivers equivalent oncological resection compared to laparoscopic approaches, with the added benefits of reduced length of stay and lower rates of conversion to open surgery. This has both clinical and healthcare economic benefits.

## Introduction

Colorectal cancer was the third most common cancer in the world in 2018, accounting for 10.2% of all new cancer diagnoses [1]. Laparoscopic surgery is a well-established technique in colorectal cancer resection. Several randomised controlled trials have demonstrated that laparoscopic surgery has similar perioperative mortality and morbidity compared to open surgery [2, 3]. The same trials have also shown that patients undergoing laparoscopic surgery benefitted from reduced intraoperative blood loss, shorter hospital stay and a reduction in analgesia requirements. Long-term outcomes in laparoscopic surgery, such as overall survival and locoregional recurrence, are comparable with open surgery [4, 5]. However, two-dimensional imaging, an unstable camera platform, limited instrumental mobility and less ergonomic freedom are some of the main limitations of laparoscopic surgery. Conversely, robotic surgery provides a stable self-controlled camera platform, with enhanced three-dimensional views, as well as instruments that have an increased range of movement, thereby overcoming many of the inherent limitations of conventional laparoscopic surgery (CLS). The advantages that robotic assisted surgery (RAS) has to offer are particularly helpful when performing total mesorectal excision dissection in a narrow pelvis. Several studies comparing CLS and RAS techniques have found no statistical difference in perioperative morbidity, bowel function recovery, conversion to open rate, or quality of oncological resection [6, 7].

One of the main drawbacks of robotic surgery is the associated cost. This includes initial capital investment, and ongoing consumable and maintenance expenses. Pai et al. reported RAS as having a higher hospital cost compared with conventional laparoscopic surgery [8]. However, in our experience, RAS may be able to mitigate these costs by contributing to a shorter hospital stay, fewer complications, and good oncological outcomes. This study aims to compare outcomes between RAS and CLS, in both left colonic and rectal resection.

## Methods

### Study population

The study population included patients who had either left sided, or rectal, cancer resections. In this study a left sided colonic resection was defined as a procedure for tumours at or below the splenic flexure, but above the peritoneal reflexion. This was recorded as a high anterior resection (HAR). A rectal cancer resection was defined as a procedure for tumours below the peritoneal reflection and total mesorectal excision was performed. This was recorded as a low anterior resection (LAR). All patients who had a primary anastomosis, with or without a defunctioning loop ileostomy, were selected for the study. At our institution, we only follow principles of Enhanced recovery after surgery [9]. However, we do not have formal programme with dedicated team.

### Data collection

Data were analysed from prospectively maintained database and online hospital databases from July 2017 to December 2020 using a variety of electronic resources and clinical notes. Data identified included patient demographics such as gender, age at the time of surgery, patient comorbidity assessment, using the American Society for Anesthesiology (ASA) grading, and Body Mass Index (BMI). Specific surgical data were also identified; this included the surgical approach used (laparoscopic or robotic), type of procedure, conversion to open, length of stay, perioperative morbidity, as well as complication data using the Clavien–Dindo score. Oncological variables, such as tumour staging (TNM), the height of tumour from the anal verge, and use of neo-adjuvant and adjuvant chemo-radiotherapy, were also recorded. Robotic surgeries were performed by three colorectal surgeons, and laparoscopic surgeries were performed by six colorectal surgeons.

The primary outcome of the study was assessment of length of hospital stay in robotic versus laparoscopic approaches. Secondary outcomes of the study identified complication rates, frequency of conversion to open surgery, and cost-effectiveness of each surgical modality.

### Data validation and statistical analyses

Data validation was carried out using a computer-generated random selection of 10% of the cases to be reviewed. All variables were analysed for normalcy, and non-parametric data were reported as medians with their accompanying Interquartile range (IQR). Categorical data were compared using Chi-squared and Kruskal–Wallis analyses. Variables which demonstrated a *p* < 0.05 were deemed statistically significant. All statistical analyses were carried out using Statistical Package for the Social Sciences (SPSS) IBM Version 27, 2020.

## Results

A total of 184 patients underwent high or low anterior cancer resections (Table 1), of which 63.0% (*n* = 116/184) were male. The median age at time of surgery was 68 years (IQR 60–73 years). Robotic surgery was performed in 40.2% (*n* = 74/184) of the study population; 45 patients had an HAR (60.8%), compared to the 70.9% (*n* = 78/110) who had HAR surgery in the laparoscopic group.

**Table 1:** Patient demographics, tumour characteristics and surgical outcomes across the study population

*n* = 184	*n*	%
Gender		
Female	68	37.0
Male	116	63.0
Age at surgery (years)		
18–60	50	27.2
61–70	67	36.4
> 70	67	36.4
American Society of Anaesthesiology (ASA)		
1	17	9.2
2	129	70.1
3	38	20.7
T		
T0	2	1.1
T1	22	12.0
T2	57	31.0
T3	91	49.5
T4	12	6.5
N		
N0	109	59.2
N1	61	33.2
N2	14	7.6
M		
M0	171	92.9
M1	13	7.1
Previous abdominal surgery		
No	147	79.9
Yes	37	20.1
Surgical approach		
Laparoscopic	110	59.8
Robotic	74	40.2
Operation type		
Low anterior resection	61	33.2
High anterior resection	123	66.8
Neo-adjuvant chemoradiotherapy		
No	178	96.7
Yes	6	3.3
Stoma		
No	127	69.0
Yes	57	31.0

Most patients were ASA 2 (70.1%, *n* = 129/184). Across the study population, the median BMI was 27.8 (IQR 25.0–30.8), and 20.1% (*n* = 37/184) had previous abdominal surgery. The median operative time was 315 min (IQR 270–367 min). The postoperative length of stay was a median of 4 days (IQR 3–8). In the CLS group, 27.3% (*n* = 30) were given a defunctioning ileostomy, with 39.2% (*n* = 29) in the RAS group receiving one. In the RAS arm, all LAR patients had ileostomy formation. Only three patients from the CLS LAR group (*n* = 29) did not receive an ileostomy. Additionally, one patient who had CLS HAR was given a loop ileostomy. The overall anastomotic leak rate, RAS or CLS, HAR or LAR, was 3.3% (*n* = 6/184).

### Robotic vs laparoscopic

There was no difference in terms of BMI or ASA grade between the RAS and CLS groups (Table 2). Tumour staging (TNM) was similar across both groups, as was the incidence of previous abdominal surgery (16.2% in robotic, 26.6% in laparoscopic, *p* = 0.107). There was no significant difference in defunctioning stoma rate between the RAS and CLS (*p* = 0.099).

**Table 2:** Comparison between the laparoscopic and robotic groups in terms of demographics, tumour characteristics and surgical outcomes

	Laparoscopic		Robotic		*p*
	*n*	%	*n*	%	
Gender					
Female	36	32.7	32	43.2	0.147
Male	74	67.3	42	56.8	
Age at surgery (years)					
18–60	22	20.0	28	37.8	** < 0.001**
61–70	38	34.5	29	39.2	
> 70	50	45.5	17	23.0	
American Society of Anaesthiology (ASA)					
1	9	8.2	8	10.8	0.120
2	74	67.3	55	74.3	
3	27	24.5	11	14.9	
Body mass index (BMI)					
Normal weight	27	24.5	14	18.9	0.074
Overweight	50	45.5	35	47.3	
Obesity class I (30.0–34.9)	28	25.5	21	28.4	
Obesity class II (35.0–39.9)	4	3.6	3	4.1	
Obesity class III (40.0 +)	1	0.9	1	1.4	
T					
0	2	1.8	0	0.0	0.330
1	19	17.3	3	4.1	
2	30	27.3	27	36.5	
3	49	44.5	42	56.8	
4	10	9.1	2	2.7	
N					
0	68	61.8	41	55.4	0.5559
1	32	29.1	29	39.2	
2	10	9.1	4	5.4	
M					
0	101	91.8	70	94.6	0.472
1	9	8.2	4	5.4	
Neo-adjuvant chemotherapy					
No	106	96.4	72	97.3	0.727
Yes	4	3.6	2	2.7	
Previous abdominal surgery					
No	86	78.2	62	83.8	0.107
Yes	24	21.8	12	16.2	
Defunctioning stoma					
No	80	72.7	45	60.9	0.099
Yes	30	27.3	29	39.1	

Bold values indicate statistically significant *p* values (*p* ≤ 0.05)

Operating time differed significantly between the two groups., with the RAS group having a shorter median operating time of 308 min, compared to 326 min in the CLS group (*p* = 0.019). The conversion to open rate was also significantly lower in the RAS group compared to the CLS group (0% vs 16.4%, *p* < 0.001, (Table 3). The length of stay was shorter in the RAS group (median = 3 days) compared with the CLS group (median = 5 days, *p* < 0.001). There was no difference in negative resection margin rates between the two groups. Both groups experienced similar rates of complications; the rate of anastomotic leak was 4% in RAS, and 5.5% in CLS (*p* = 0.673). Overall, there was no significant difference in complication rates between the two groups (*p* = 0.164).

**Table 3:** Comparison of outcomes between robotic and laparoscopic groups

	Laparoscopic		Robotic		*p*
	*n*	%	*n*	%	
Conversion to open					
No	92	83.6%	74	100.0%	** < 0.001**
Yes	18	16.4%	0	0.0%	
Resection margin					
R0	110	100.0%	73	98.6%	0.222
R1	0	0.0%	1	1.4%	
Anastomotic leak					
No	104	94.5%	74	100.0%	**0.673**
Yes	6	5.5%	3	4.0%	
Complications					
No	88	80.0%	65	87.8%	0.164
Yes	22	20.0%	9	12.2%	
Length of stay					
	Median 5 days	IQR 4–9 days	Median 3 days	IQR 3–5 days	** < 0.001**
Operating time					
	Median 326 min	IQR 281–376 min	Median 308 min	IQR 238–356 min	**0.019**

Bold values indicate statistically significant *p* values (*p* ≤ 0.05)

### Predictors of poor outcome

Univariable and multivariable binary logistic regression analyses were carried out to determine factors associated with poor outcomes. Poor outcome was defined as an anastomotic leak, R1 resection (positive resection margin), and a length of stay of 5 days or longer. Given the low number of anastomotic leaks (*n* = 9) and R1 resections (*n* = 1), regression analyses were not possible for either of these two outcomes. However, multivariable analysis was performed to determine factors associated with prolonged length of stay, defined as more than 5 days, (Table 4).

**Table 4:** Univariable and multivariable logistic regression analyses comparing laparoscopic and robotic groups

	Univariable	Multivariable		
		OR		*p*
Gender				
Female	0.136	1 (Reference)		
Male				
Age at surgery (years)				
18–60	**0.004**	1 (Reference)		
61–70		0.52	0.22–1.25	0.141
> 70		2.00	0.86–4.67	0.109
ASA				
1	**0.033**	1 (Reference)		
2		1.33	0.38–4.64	0.655
3		1.83	0.40–8.32	0.435
BMI				
Normal weight	0.800			
Overweight				
Obesity class I (30.0–34.9)				
Obesity class II (35.0–39.9)				
Obesity class III (40.0 +)				
Missing data				
T				
0	0.587			
1				
2				
3				
4				
N				
0	0.807			
1				
2				
M				
0	0.682			
1				
Surgical approach				
No	** < 0.001**	1 (Reference)		
Yes		0.24	0.18–0.49	** < 0.001**
Previous abdominal surgery				
No	0.497			
Yes				
Post-operative complications				
No	** < 0.001**	1 (Reference)		
Yes		10.33	3.32–32.10	** < 0.001**

Bold values indicate statistically significant *p* values (*p* ≤ 0.05)

Univariable analysis revealed that age, ASA grade, surgical approach, and the presence of postoperative complications, were associated with an increased length of stay. These variables were entered into a multivariable model. Following multivariable analysis, surgical approach and postoperative complications were independently associated with length of stay. Patients who underwent RAS were significantly less likely to stay 5 days or more (OR 0.20, 95% CI 0.08–0.40, *p* < 0.001) compared with CLS. Patients who experienced a complication had more than a tenfold increased risk of requiring a longer length of stay (OR 10.01, 95% CI 3.09–32.32, *p* < 0.001). Undergoing a low anterior resection was also independently associated with a longer length of stay (OR 3.37, 95% CI 1.54–7.36, *p* = 0.002).

## Discussion

The pertinent finding of this study is that RAS can significantly reduce postoperative length of hospital stay. Additionally, and within colorectal surgery, RAS can reduce the need of converting to an open procedure compared to CLS. All patients within the study had similar post-operative care programme following the majority of principles of enhanced recovery after surgery. This included early mobilisation, adequate pain management (patient-controlled analgesia, epidural and oral analgesia), and commencement of an oral diet from post-operative day 0. Additionally, all HAR patients in the RAS group had their catheter removed on post-operative day 1. Importantly, RAS achieved similar oncological outcomes to CLS, with no significant difference in resection margin positivity. The R1 resection, reported in the RAS group was due to encapsulated lymph node metastasis close to the circumferential resection margin (CRM). These findings are in keeping with two recently published meta-analyses comparing RAS to CLS [10, 11], and which demonstrated that shorter hospital stay and lower conversion rates.

A 2018 meta-analysis by Prete et al. [11], compared 334 robotic rectal resections with 337 laparoscopic rectal resections, across five different trials. The RAS group demonstrated similar oncological (lymph node yield and margin clearance) and surgical safety (30-day mortality) compared to the CLS group. Additionally, RAS showed a lower conversion rate, but demonstrated longer operative duration compared to laparoscopic surgery. Another meta-analysis, conducted by Ng et al. [10], showed that RAS has a statistically significant advantage over CLS in conversion rates, wound infections, all-cause mortality and duration of hospital stay in colorectal cancer resections. Contrary to both of these studies, we found our operative times to be shorter in the RAS group compared to the CLS arm. However, an explanation for this might be that the majority of CLS operations were either partly, or fully performed by trainee registrars under consultant supervision, whereas RAS resections were primarily performed (console surgery) by consultant surgeons but the open components (colonic conduit preparation, stoma creation, closures of the wounds, etc.) were still performed by the trainee surgeons. This may also explain why our colorectal unit were able to perform two robotic colorectal resections a day, and thus replicating a standard CLS day theatre session. Nevertheless, our unit managed to maintain the same level of theatre utilisation with good productivity.

Some of the key advantages of RAS over CLS include improved ergonomics, high fidelity reproduction of human hand movements, a high-definition 3D camera system, stable platform, tremor eliminator and EndoWrist^®^ technology. These features provide better access to regions of the body, such as the pelvis, where reticulation/rotation and generalised movements of instruments are often restricted owing to the restrictive nature of the cavity. Improved access to difficult to reach regions may explain the relatively low conversion rate within the RAS group (0% in RAS vs 16.4% in CLS). A conversion rate of 16.4% is higher than the national UK average of 8%, as quoted in the National Bowel Cancer Audit (NBOCA) [12]. However, within our study, only left-sided and rectal cancer resections (HAR and LAR) have been included, and these procedures typically have a higher rate of conversion. Meta-analysis data appear to corroborate our conversion rate, reporting a 1–7.3% conversion rate in RAS [13]. Potential reasons for conversion from laparoscopic to open surgery include difficulty in accessing the target organ and higher BMI [14], [15]. These limiting factors can be mitigated in RAS due to the technological advantages possessed by the robot. Hence the conversion rates are lower in RAS, even in rectal resections [16].

The ROLARR trial [6] failed to show any significant difference between CLS and RAS outcomes, although RAS did have a lower conversion rate (12.2% vs 8.1%). These findings have been widely debated because the experience of surgeons in each arm was significantly different; CLS and RAS surgeons had an average of 91 and 50 cases respectively. This was considered as a major influencing factor causing potential bias, i.e., conversion rates were higher because surgeons using the robot had less experience with this operative modality.

One of the main criticisms of RAS is its higher cost per patient [6]. In the ROLARR trial, it was estimated that each RAS patient attracts a fixed cost of $1611 per procedure, in addition to variable costs, such as consumables. However, it is possible for these costs to be mitigated up to a certain extent. For example, at our unit (Norfolk and Norwich University Hospital), we tend to perform a 3-arm high anterior resection rather than the standard 4-arm, thereby saving £187 per procedure. We have also avoid using single-use equipment, such as vessel sealers (as it adds value in very high BMI patients), to further reduce costs but without any compromise to outcome, or duration of surgery. The recently introduced ‘Intuitive extended use programme’ has significantly reduced the unit cost of commonly used instruments by increasing their life cycles. For instance, Cadiere forceps now have 18 cycles of use, compared to 10 cycles, which helps to reduce the cost per use from £187 to £116. With competition from other manufacturers coming into play, the unit cost for RAS is likely to reduce even further in the future (Appendix 1).

A significantly reduced length of stay and lower morbidity in the RAS group is also likely to reduce costs further. According to the Health and Social Integration document, released by the national audit office in 2015–16, an elective bed costs the NHS approximately £306 per day [17]. However, this estimation climbs steeply when adding in surgical care and other interventions such as physiotherapy, nursing care, catering, medication, etc. On average, our RAS patients have a length of stay 2 days shorter than our CLS patients. This not only reduces the cost to the NHS, but it also facilitates bed capacity for elective and non-elective surgical services. In the current COVID-19 climate, which has caused a significant elective surgical backlog, shorter post-operative stay would be expected to facilitate improved efficiency of the NHS.

**Fig. 1:**
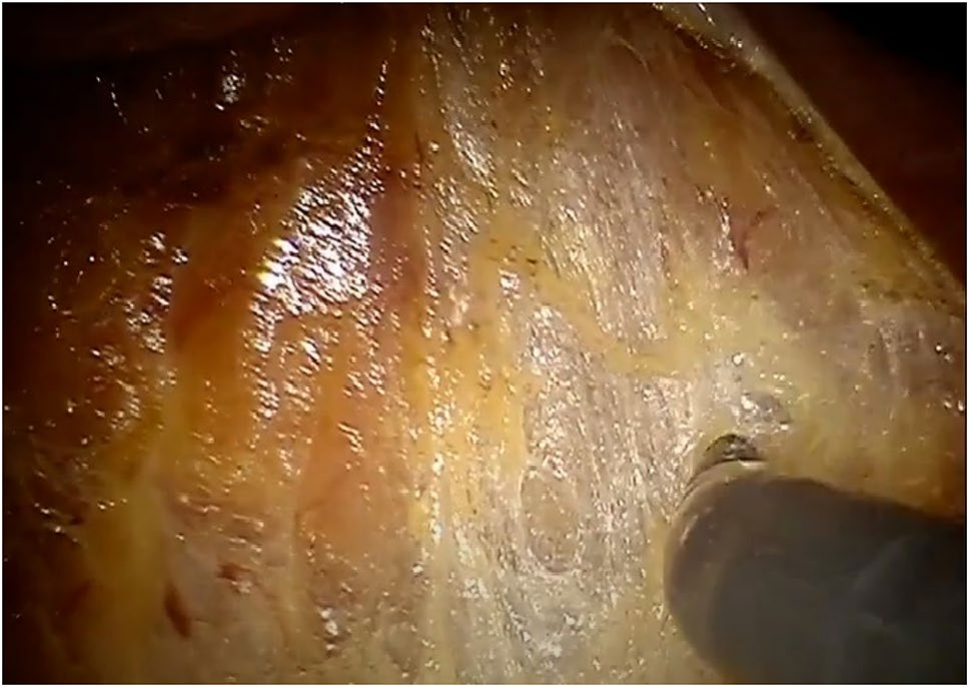
Mesorectal dissection with enhanced view

**Fig. 2:**
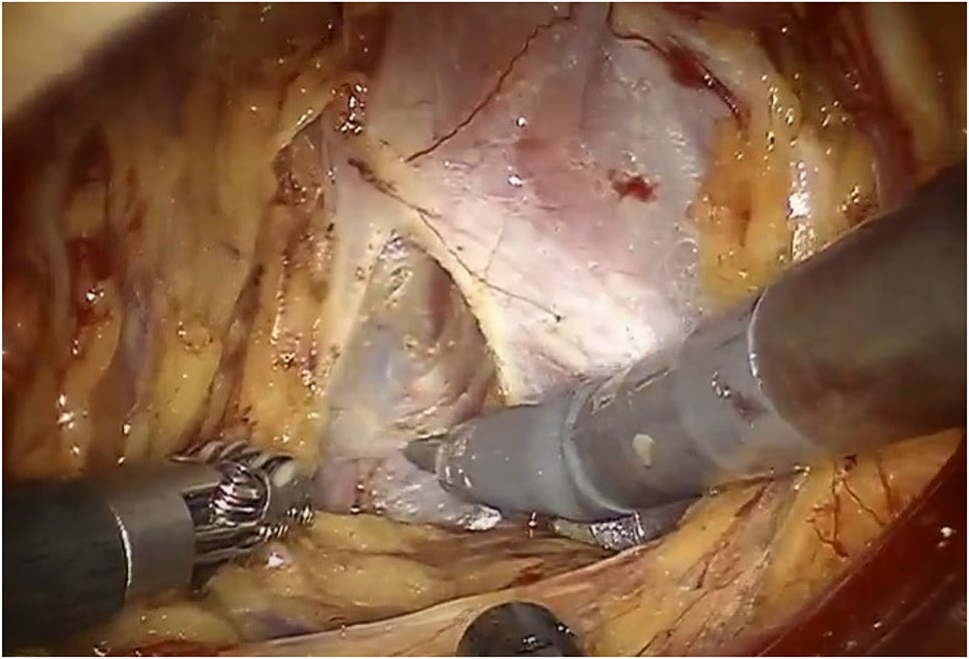
Pelvic floor dissection

The robotic surgeries included within this study were performed using either a da Vinci^®^ Si or a da Vinci^®^ X system. One of the drawbacks in the older, Si generation was difficulty in accessing two different operative fields (pelvis for rectum and left upper quadrant for splenic flexure) without having to change port configurations intraoperatively. This no doubt contributed to longer operating times. However, the newer systems, da Vinci^®^ X and Xi^®^, have a lot more setup flexibility, which has gone someway to mitigating the issue of redocking, thus operating time can be reduced with the use of these two systems (Figs. 1, 2).

There is also evidence in the literature that colorectal fellows being trained in robotic colorectal resections is safe and does not negatively impact on the oncological and surgical quality of the procedure [18]. It is therefore imperative that a national or indeed international drive is undertaken to ensure that a new generation of would-be surgeons are trained in robotic colorectal surgery to disseminate robotic surgery, much in the same way that paved the way for laparoscopic colorectal surgery becoming the norm in the earlier part of this century. Our robotic fellowship programme started in October 2021. We are hoping to compare the outcomes in coming years.

### Limitations of the study

The main limitations of this study are that this is a single centre, retrospective analysis of the data. Although the two groups appeared evenly matched across key variables, patients are subjected to a certain element of case selection. Furthermore, we accept that the data presented in this study may not necessarily be reflective of practice elsewhere. Procedure-wise, RAS was performed by primarily three experienced consultant colorectal surgeons whilst CLS was performed by a combination of six experienced colorectal consultants which include RAS performing surgeons. Trainee surgeons were performing surgeries in both groups, but console surgery primarily performed by consultants in RAS.

Right sided operations were not included in this study due to discrepancies between RAS and CLS groups, as most RAS patients underwent complete mesocolic excision as opposed to those within the CLS group who underwent standard right hemicolectomy. Abdominoperineal resections were also omitted due to their lack of primary anastomosis and the very small number within the robotic arm, rendering statistical analysis unsuitable. Therefore, the results are strictly limited to left-sided colonic cancer resections with primary anastomosis.

Furthermore, for the purposes of statistical analyses, we have had to combine groups together (i.e., left sided/sigmoid resections combined with rectal cancer) and we accept that clinically this represents a heterogenous group of patients who may have different risks/tumour biology.

## Conclusion

RAS provides a similar standard of oncological resection to CLS in colorectal cancer and may even be advantageous over the CLS in terms of RAS’s reduced length of stay, and reduction in conversion to open surgery. With the advent of further robots, as well as a general trend towards cost reduction, further future randomised trials would be useful in assessing whether robotic surgery is likely to supersede laparoscopy in left colonic and rectal cancer surgery.
